# Recurrent development of song idiosyncrasy without auditory inputs in the canary, an open-ended vocal learner

**DOI:** 10.1038/s41598-018-27046-4

**Published:** 2018-06-07

**Authors:** Chihiro Mori, Wan-chun Liu, Kazuhiro Wada

**Affiliations:** 1 Graduate School of Life Science, Hokkaido University, Sapporo, Hokkaido Japan; 2 Department of Biological Sciences, Hokkaido University, Sapporo, Hokkaido Japan; 3 Faculty of Science, Hokkaido University, Sapporo, Hokkaido Japan; 4 Department of Psychology, Colgate University, Hamilton, New York USA; 5 Present Address: Department of Life Sciences, Graduate School of Arts and Sciences, The University of Tokyo, Tokyo, Japan

## Abstract

Complex learned behaviors, like bird song and human speech, develop under the influence of both genetic and environmental factors. Accordingly, learned behaviors comprise species specificity and individual variability. Auditory information plays a critical role in vocal learning by songbirds, both to memorize tutor songs and to monitor own vocalizations. Nevertheless, audition-deprived songbirds develop structured, species-specific song patterns. It remains to be elucidated how the auditory input contributes to the development of individual variability of song characteristics. Here we show that an open-ended vocal learner, the canary, annually recapitulates individually unique songs without audition. Although the total number of syllable types was reduced by auditory deprivation, other vocal phenotypes examined in the syllable, phrase, and syntax of songs were conserved between the 1^st^ and 2^nd^ years, both in deafened and intact birds. In deafened canaries, approximately 60% of the syllables were yearly reproduced with consistent acoustic features, whereas the remaining syllables were replaced with new ones in an annual cycle of song development. These results indicate that the open-ended vocal learning of canaries involves an audition-independent mechanism for the development of recurrent song idiosyncrasy.

## Introduction

**Figure 1:**
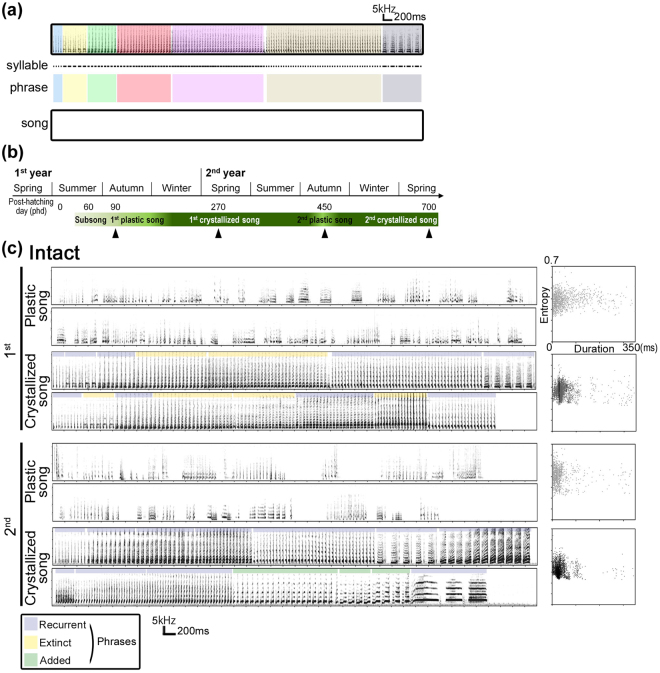
Song structure and annual cycle of song development in the canary. (**a**) Syllables and phrases in a canary song. The black dots under the sonogram represent the syllables, which are the smallest song elements. Repetitive syllables constitute a phrase, which is indicated by color shading. (**b**) The annual cycle of song development in the canary. Arrowheads represent the time points of song analyses used in this study. (**c**) Examples of songs and syllable scatter plots (x-axis: duration, y-axis: entropy) from an intact canary. Representative sonograms during instability and crystallization of songs in the 1^st^ and 2^nd^ years. Plastic songs were recorded in the fall (from September to October). Crystallized songs were recorded in the spring (from March to June). Phrases are indicated by color lines. Blue lines represent “recurrent” syllable phrasing that appears in crystallized songs in both the 1^st^ and 2^nd^ years. Yellow lines represent “extinct” syllable phrases that appear only during the 1^st^ year. Green lines show “added” syllable phrases that appear newly in the 2^nd^ year.

Birdsong, like human speech, is a form of learned vocalization^1,2^, which serves two important communicative functions: to signal species specificity and individual identity^3–6^. Songbirds learn syllable acoustics and sequential features from conspecific tutor songs^7^. Therefore, audition is a crucial sensory input to develop functional vocalizations, memorize the tutor song, and monitor own vocal outputs. However, juvenile songbirds do not develop their songs from a “blank slate”-like state of extensive plasticity^8^. Even when deprived of audition before hearing tutor songs, juvenile birds later develop species-specific songs with a certain degree of structure and individual uniqueness^9–14^. Previous studies suggest that an intrinsic program *per se* ensures an effective competency for self-differentiation of structured song patterns. However, it remains to be understood how the individual uniqueness of specific song features is generated independently from audition and alongside the maintenance of species-specificity. We investigated this question in an open-ended vocal learner, the canary, which annually acquires a set of new song elements, even throughout adulthood^15–18^. The song development by the canary have a hierarchical species-specific structure, composed of syllables and phrases (*i.e*., clusters of repetitive syllables)^19^ (Fig. 1a). During the 1^st^ post-hatching year, juvenile males start singing subsong in the summer. The song pattern is crystallized until late winter, and the birds continue to sing it until the following summer (Fig. 1b,c). By the fall of the 2^nd^ year, their songs deteriorate to a degree of subsong/early-plastic song form, characterized by acoustically amorphous syllables and unstable sequences, after which new songs are re-crystallized by the 2^nd^ year’s late winter^20,21^. During the annual song development, three categorical types of syllables, which we termed recurrent, extinct, and added syllables, are identifiable by comparing the crystallized song of the 1^st^ and 2^nd^ years. Taking advantage of this annual syllable subset exchange, we examined whether and how song syllables were recurrent/replaced without auditory input, to investigate the developmental recurrence of species specificity and individual uniqueness of complex vocalizations in the same individual.

In the present study, we deafened individuals of an inbred canary strain, the Belgian Waterslager, at an early developmental stage during the 1^st^ post-hatching year, and recorded their songs until song crystallization in the 2^nd^ year. We examined the similarities and differences in the hierarchical structures of their songs between the 1^st^ and 2^nd^ years and between intact and deafened birds. Despite drastic changes in the syllable phrasing during the annual cycle of song development, deafened canaries iteratively generated species-specific songs with consistent individual uniqueness. This result reveals the existence of an auditory-independent innate program to generate and retain individual uniqueness within a species-specific song pattern.

## Results

### Annually repeated song crystallization and deterioration in the deafened canary

**Figure 2:**
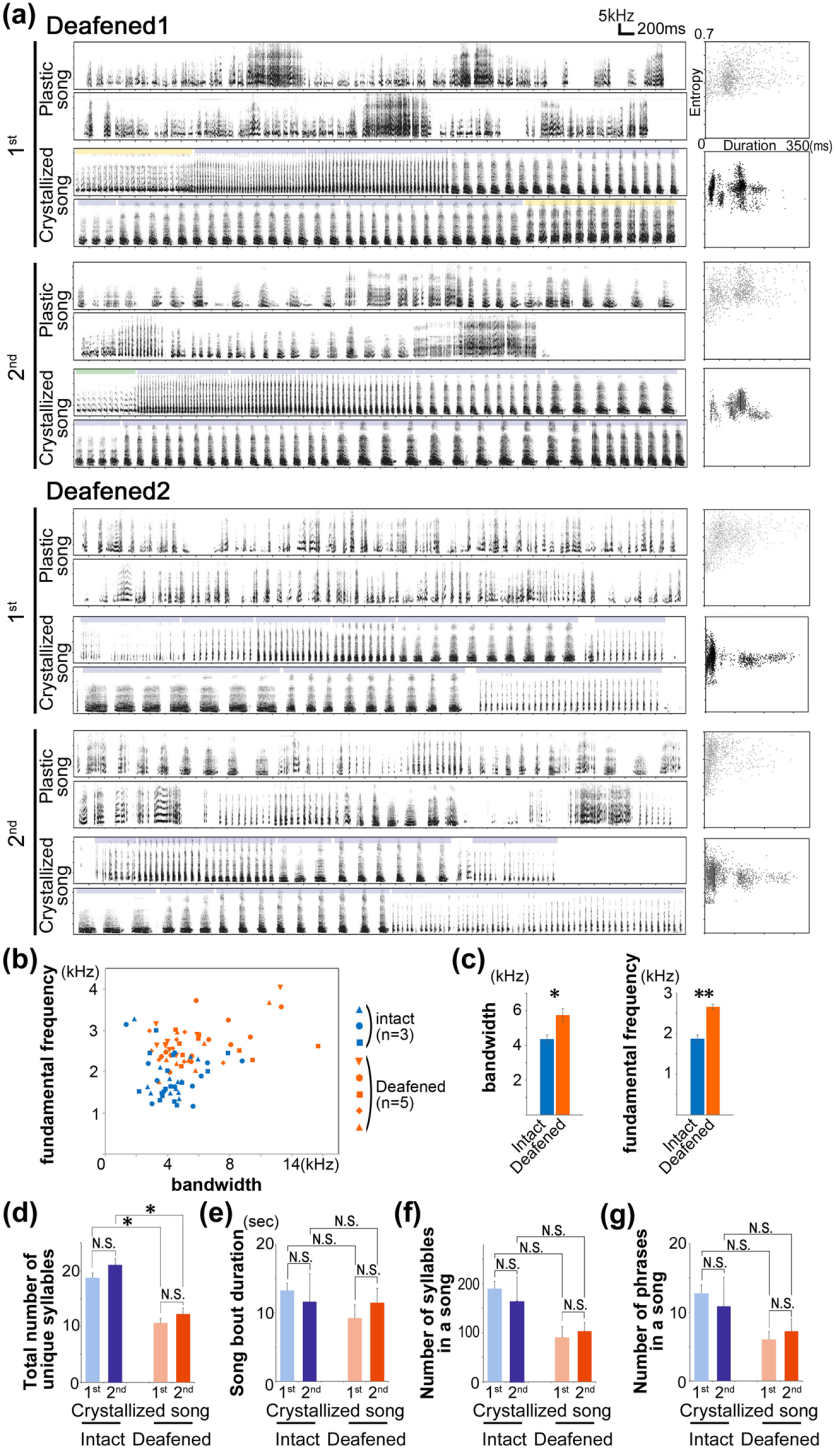
Annual song crystallization and deterioration in deafened canaries. (**a**) Examples of songs and syllable scatter plots (x-axis: duration, y-axis: entropy) from two deafened canaries. Representative sonograms showing plastic and crystallized song stages in the 1^st^ and 2^nd^ years. (**b**) Comparison of syllable bandwidth and fundamental frequency between intact and deafened canaries (n = 3 and 5 birds, respectively). Each dot indicates the average of each syllable type (20 syllables per a syllable type including recurrent, added, and extinct). There are 8–15 syllable types per bird. (**c**) Mean syllable bandwidth and fundamental frequency for intact and deafened birds. ^*^P < 0.05, ^**^P < 0.01; *t*-test after Holm’s correction. Error bars: SEM. (**d**–**g**) Total number of unique syllables (**d**), song bout duration (**e**), number of syllables in a song (**f**), and number of phrases in a song (**g**) in the 1^st^ and 2^nd^ years for intact and deafened birds. ^*^P < 0.05; *t*-test after Holm’s correction. Error bars: SEM.

To examine the effects of auditory deprivation on the annual development and retention of the individually unique canary songs, juvenile males were deafened through bilateral cochleae removal, before fledging at 27–34 post-hatching day (phd). This procedure ensured the loss of auditory experience, depriving the canaries from both the tutor song and their own vocal output. As previously reported^13,14^, auditory deprivation significantly affected the acquisition of syllable acoustic features in the used canary strain (Fig. 2a–c). However, the annual song development, that is, the timing of song crystallization and successive deterioration, was similarly regulated in both deafened and intact birds. Both deafened and intact canaries produced plastic songs by the fall and crystallized them from winter to spring (Figs 1c and 2a). Moreover, the crystallized song patterns of both deafened and intact birds consisted of a series of repertoires with phrases composed of repetitive syllables. However, the crystallized songs of deafened birds included approximately half the number of syllable types than those of intact birds both for the 1^st^ and 2^nd^ years (P^*^ = 0.011 and 0.004 in the 1^st^ and 2^nd^ years, respectively; Student’s *t*-test with Holm’s correction) (Fig. 2d), although no significant differences were found between the 1^st^ and 2^nd^ years and between deafened and intact canaries regarding song bout duration and the number of syllables or phrases in a song (Fig. 2e–g**)**. These results indicate that the annual timings of song crystallization and deterioration were regulated independently from the auditory input. Furthermore, the fundamental structure of the canary song was also constructed in an auditory-independent way.

### Recurrent intrinsic unique syllable phrasing in deafened canary

**Figure 3:**
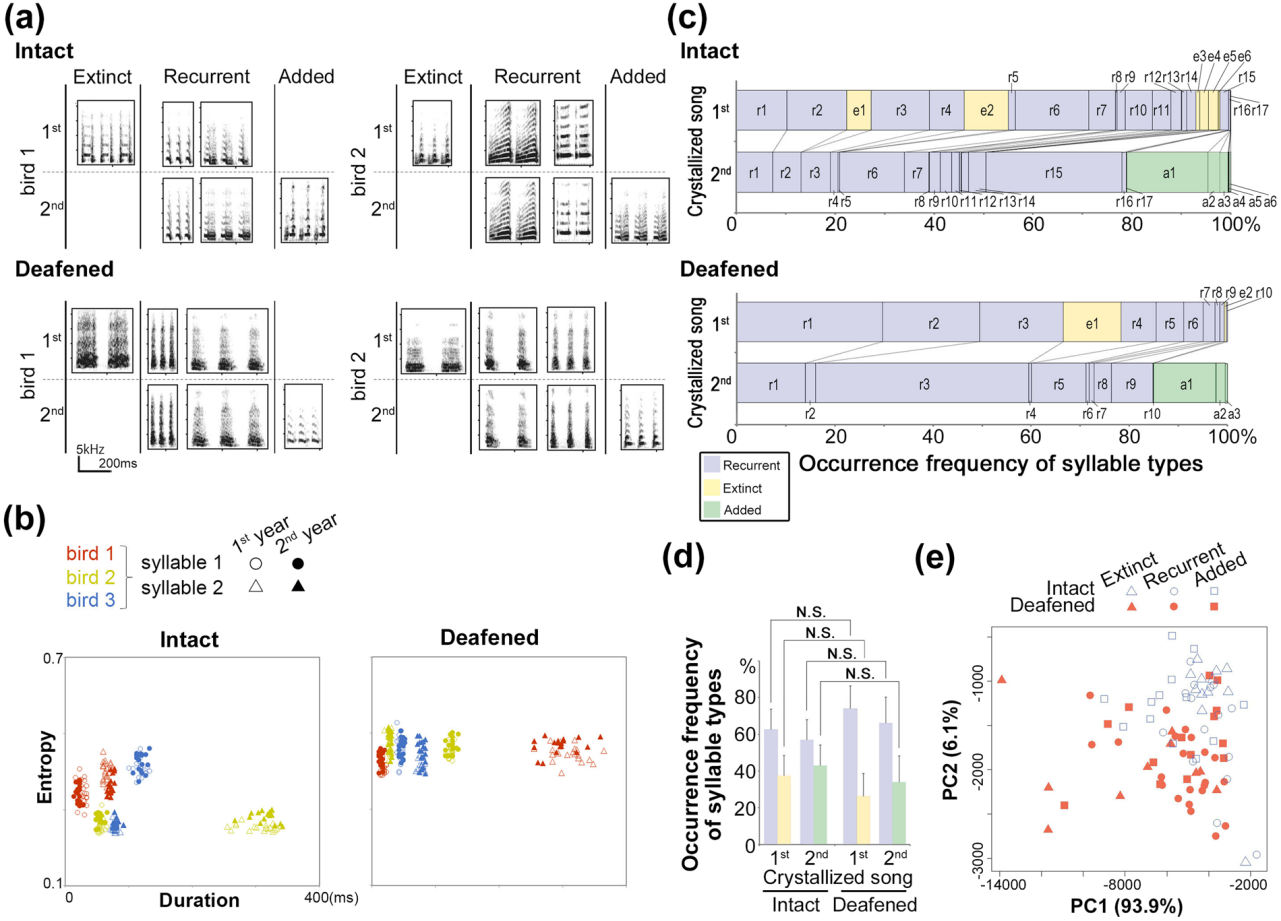
Auditory-independent regulation of phrase repertoires between the 1^st^ and 2^nd^ years. (**a**) Examples of recurrent, extinct, and added syllables of two intact and deafened canaries. (**b**) Recurrence of individual differences of syllable acoustics in intact and deafened canaries. Scatter plots of recurrent syllables (average of 20 syllables per a phrase) from intact and deafened canaries (n = 3 birds each; two example syllables from a bird). Open and closed symbols indicate syllables in the 1^st^ and 2^nd^ years, respectively. (**c**) Examples of proportion of syllable types in an intact and a deafened bird. The alphabet indicates phrase types (*e.g*., “r,” “e,” and “a” represent recurrent, extinct, and added syllable types, respectively). (**d**) Frequency of occurrence per syllable type. Recurrent, extinct, and added syllables are colored with blue, yellow, and green, respectively. Error bars: SEM. (**e**) Principal component analysis using syllable acoustic parameters on phrase types in intact and deafened birds. Each dot indicates a syllable type.

**Figure 4:**
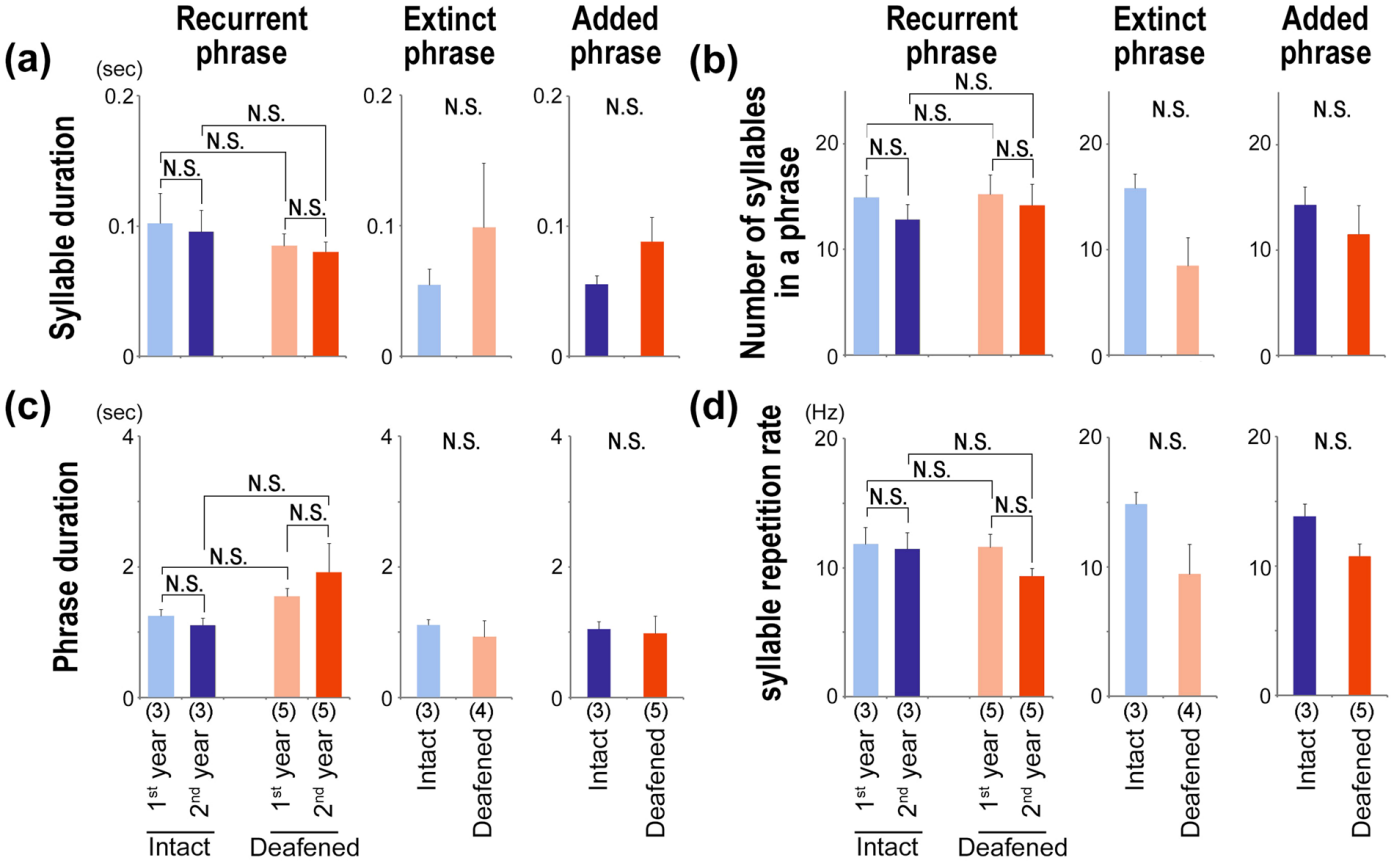
Conserved temporal phenotypes of phrases between intact and deafened canaries. Syllable duration (**a**), number of syllables in a phrase (**b**), phrase duration (**c**), and syllable repetition rate (**d**). Number of animals in each group is indicated in brackets at the bottom of bar graphs. Error bars: SEM.

We then compared the acoustic and temporal features of the syllables and phrases in crystallized songs, between the 1^st^ and 2^nd^ years and between intact and deafened birds. On the basis of the acoustic features and inter-syllable gap timing shown in the spectrogram, we identified three types of syllables, present in the songs of both deafened and intact canaries: extinct, recurrent, and added syllables (Fig. 3a). Individual differences in the acoustic features of recurrent syllables were maintained and reproduced yearly without auditory input in both deafened and intact birds (Fig. 3b). After song deterioration in the 2^nd^ year, both deafened and intact birds recapitulated approximately 60% of the individually unique syllables as recurrent syllables in their new crystallized songs (Fig. 3c,d). This indicates that individually unique syllable variations are retained in an auditory-independent manner. The frequency of occurrence of extinct, recurrent, and added syllables was conserved between intact and deafened birds (Fig. 3d). In addition, during annual song development, the syllable repertoires were reassembled through the loss of some syllables (extinct syllables) and addition of new ones (added syllables), resulting in the maintenance of a similar proportion (approximately 30–40%) in songs between the 1^st^ and 2^nd^ years, even in deafened birds (Fig. 3d). These results indicate that an intrinsic, audition-independent genetic program is necessary and sufficient to regulate syllable maintenance and replacement. Principal component analysis (PCA) using five acoustic syllable parameters: duration, inter-syllable interval, fundamental frequency, bandwidth, and entropy, revealed the absence of a clear boundary between the three syllable categories (Fig. 3e). The acoustic features of the syllables in the PCA were mostly merged between intact and deafened birds. In line with this finding, the syllable duration, phrase duration, and number of syllables in a phrase did not differ significantly between deafened and intact birds for all three categorized types (Fig. 4a–c). In addition, although it has previously been reported that the syllable repetition rate is reduced in socially isolated canaries^22^, in the present study no significant difference was observed in the syllable repetition rates between deafened and intact birds, when comparing recurrent, extinct, and added phrase types separately (Fig. 4d). Taken together these results suggest that, although the total number of acquired unique syllables in crystallized songs was decreased by auditory deprivation (Fig. 2d), the frequency of occurrence and temporal features of the three categorized types of syllable phrasings were intrinsically regulated.

### Auditory-independent song syntax in the canary

**Figure 5:**
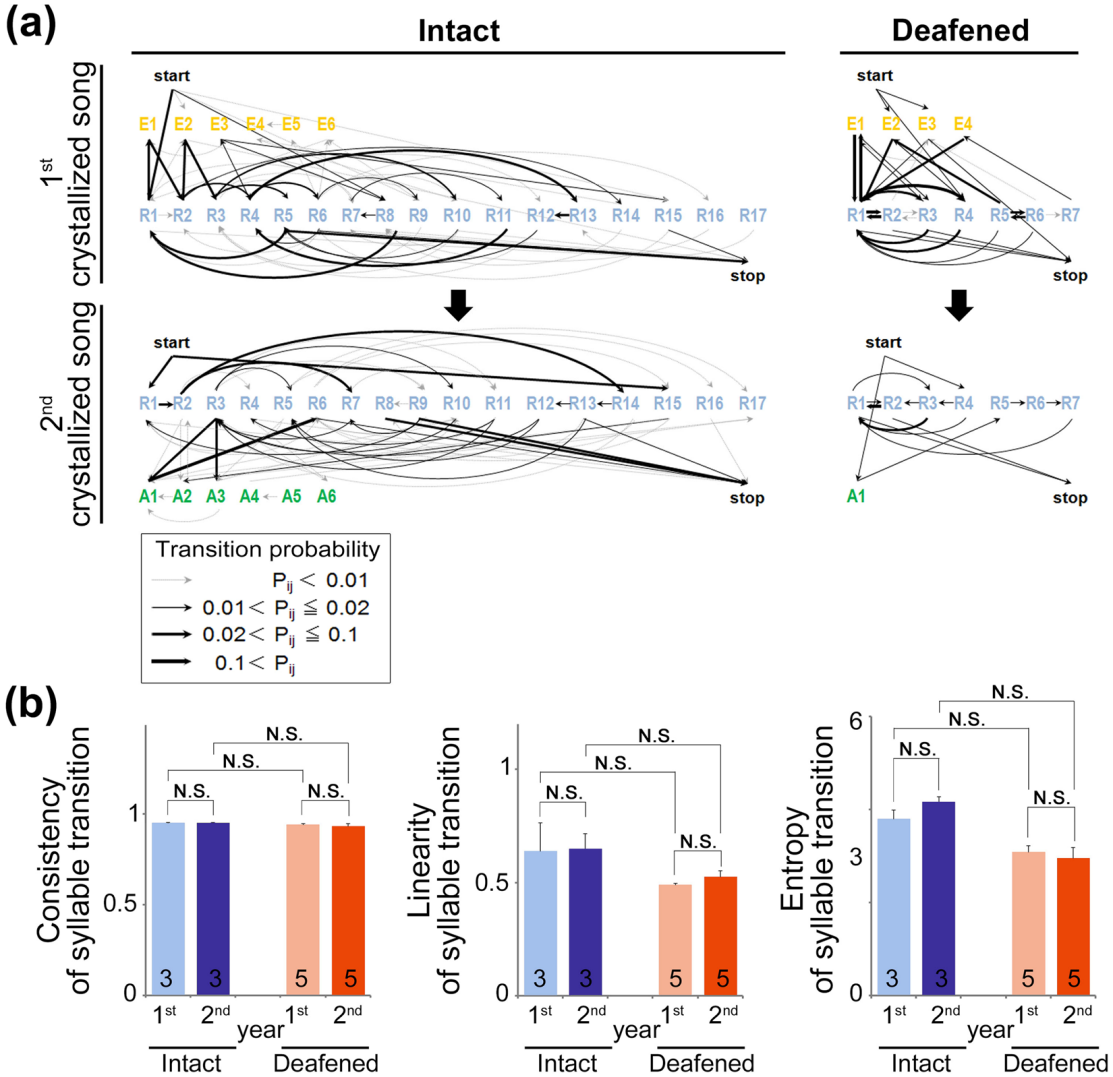
Auditory-independent regulation of song syntax in the canary. (**a**) Examples of phrase transitions in the 1^st^ and 2^nd^ years in intact and deafened canaries. In phrase transition diagrams, the probability (P_*ij*_) of the transition from phrase *i* to phrase *j* is represented by line thickness (higher P_*ij*_, thicker line). (**b**) Comparison of mean consistency, linearity, and entropy of syllable transitions in the 1^st^ and 2^nd^ years between intact and deafened birds. The number of animals within each group is indicated by inside bars. Error bars: SEM.

We next examined the effect of auditory deprivation on song syntax in the canary. Variations in song phrase transitions indicated a decreasing trend in deafened birds compared with intact birds, as a reduction of the total number of unique syllables was observed following auditory deprivation (Fig. 5a). Although the phrase repertoires were not randomly generated, the probability of transition among phrases, even phrases composed of recurrent syllables, was highly variable in both intact and deafened canaries (Fig. 5a). In addition, the probability of transition among phrases changed between the 1^st^ and 2^nd^ years. These results indicate that phrase ordering was audition-independently regulated and annually reorganized. For quantitative analyses of song syntax phenotypes in the canary, we calculated consistency, linearity, and entropy of syllable transitions in songs. No significant differences were found in the examined phenotypes of song syntax between intact and deafened birds and between the 1^st^ and 2^nd^ years’ crystallized songs (Fig. 5b). This result indicates an auditory-independent recurrence of species-specific song syntax in the canary, which is similar to that observed in the regulation of acoustic and temporal features of syllables and phrases.

## Discussion

Previous studies indicate that the canary develops a typical, species-specific song structure, which is strictly constrained by an innate predisposition regardless of song tutoring conditions (*e.g*., isolation or artificially synthesized song)^18,23,24^. In the present study, we used early-deafened canaries and showed the intrinsic, auditory-independent repeatability in the generation of an individually unique, species-specific song structure. The Waterslager canary strain has been previously reported to show impaired high-frequency sound hearing associated with inherited reduction of hair cells^25–27^ and a lower spectral distribution of energy in the vocalization, compared with other canary strains^28^. Although the elevated thresholds at high frequencies may lead to differences in song development, compared with the wild populations or with other species, auditory deprivation clearly affected song acoustics in this strain (Fig. 2b,c)^13,18^. These facts indicate that Waterslager canaries use auditory information for song development, thus contrasting with other songbird species that develop normal songs and song repertoires without appropriate auditory experiences^29,30^. The reduction of unique syllable types caused by hearing loss was similar to that observed in canaries of the same strain raised in high noise environments, until deafened^13^. However, those canaries produced more variable syllables than the subjects of the present study^14^, suggesting that the pre-deafening auditory experience might influence the generation of syllable variability after auditory deprivation. In the present study, deafened canaries annually developed species-specific songs composing of acoustically consistent recurrent syllables. In addition, syntactical phrase ordering was audition-independently regulated and annually reorganized. During annual song development, the phrase repertoires were reconstructed by replacing extinct syllables with newly added ones, with no auditory input. This suggests that a predisposed innate program is crucial for the regulation of the annual syllable maintenance and replacement, which may comprise the behavioral basis for open-ended learning.

The canary annually repeats song crystallization and deterioration during adulthood, which is a characteristic of open-ended vocal learning. After song deterioration with the collapse of acquired syllable acoustics and sequential features, the song is re-crystallized maintaining recurrent syllables, expressed in the previous crystallized songs. Song acquisition proceeds by trial-and-error sensorimotor learning to approximate a tutored song^31–33^. Therefore, such recurrence of acoustically and temporally conserved syllables would be feasible through auditory experiences, both for memorizing the song template and for monitoring the bird’s own vocal outputs. However, our study using early-deafened canaries contradicts this perspective by showing an audition-independent production of recurrent song syllables. This result indicates that, rather than auditory instruction, the generation and maintenance of individual uniqueness in canary song depends on predisposed genetic or other epigenetic programs. Although the proprioceptive feedback from the syrinx and/or respiratory organs may be a contributing factor to guide song development in a deafened condition^7,12,34^, production of consistent acoustic features of syllables can only be achieved by precise regulation of complex high-dimensional parameters, coordinating multiple syrinx muscles^35^. Therefore, rather than random, strict neuronal connections should be recurrently reconstructed with reference to intrinsic information during annual song development. A large fraction of the synaptic connections between the HVC and the robust nucleus of the arcopallium RA neurons is replaced each year from spring to fall in adult canaries^36,37^. However, a subpopulation of these connections and most projections from the HVC to Area X could be persistently retained through an annual cycle of song development^38–40^. The persistent connections of HVC neurons projecting into the RA and Area X might contribute to the reproduction of consistent acoustic and temporal features of syllables without auditory input. In addition, the replacement of new HVC neurons, which is regulated by hormones, singing, photoperiod, and neuronal activity, could be a crucial modulator of syllable addition and loss even under auditory deprivation^41^. Further research is needed to investigate how such internal and external factors influence the neural mechanisms for genetic-driven restoration of complex vocalizations.

One of our motivations for this study was to compare the song of early-deafened canaries to our previous results from a closed-ended learner, the zebra finch, which was deafened at a similar early developmental stage^42^. Although there are accumulated observations of song phenotypes in the oscine songbird species after deafening, the timing of song crystallization/stabilization without auditory input has not been fully investigated. Early-deafened zebra finches show an obvious delay of song crystallization, which takes 2- to 3-folds longer time compared with intact zebra finches^42^, thus indicating an auditory-dependent, active regulation of song crystallization. This auditory-dependent regulation of song structures is also observed in the Bengalese finch, which is also a closed-ended learner^43,44^. However, in the canary, the auditory deprivation did not affect the seasonal timing of song crystallization. These results clearly demonstrate that the auditory contribution to crystallization of song structures differs across species. Further comparative experiments are needed to evaluate how the auditory experience directly affects the regulation of song crystallization and plasticity.

Auditory experience enhances an individual uniqueness to acquire a large number of unique and sexy syllable types, hence successfully attracting females^45^. However, individual variability in song generation is partly intrinsically prepositioned in the canary. The degree of expression of auditory-independent individual variability in song structure was also different between the zebra finch and the canary. Deafened zebra finches show a wider range of individual differences in both syllable acoustics and the sequential structures of their crystallized songs compared with that of intact birds^42^. This individual diversification increases among different families. In contrast, deafened canaries mainly exhibited individual uniqueness at syllable acoustics, not at syllable phrasing and transition sequence. Unlike zebra finches, auditory deprivation did not affect individual variation in the canary. This species-specific constraint for expression of individual variation would be reflected by the history of selective breeding of canaries, on the basis of the song phenotype. Although no selective breeding has been performed in the zebra finch, the canary has a long history spanning over 500 years of selection based on their song features, especially regarding frequency modulation of syllables and song length^19,46^. Therefore, the degree of genetic variation among individuals should be different between the two species, that is, much less variability of genetic diversity in the canary than the zebra finch. This limited genetic variation caused by inbreeding may be related to the intrinsic origin of the strict constraint in species-specific song structure, suggesting a genetic reduction of the plasticity of individual variability in song learning.

## Methods

### Animals

We used an inbred canary strain, the Belgian Waterslager, housed at the Rockefeller University Field Research Center. The birds were raised in individual breeding cages with their parents and siblings until the 27^th^ to the 33^rd^ phd. By fledging, the sex of birds was checked by PCR as previously reported^47^. We set eight male juveniles into two groups: auditory-intact (n = 3) and deafened (n = 5). All subjects within each group were housed together with other auditory-intact birds. The photoperiod corresponded to the annual light cycle of the New York State, USA. Food and water were available *ad libitum* throughout the year. All animal experiments were performed in accordance with a protocol approved by the Rockefeller University Institutional Animal Care and Use Committee.

### Deafening

The deafening operation was performed as previously described^42^. Five males were deafened by bilateral cochlear extirpation at 27–34 phd (average = 29.8 phd). The birds were anesthetized with Nembutal administered by intraperitoneal injection. After fixing the head in a custom-made stereotaxic apparatus with ear bars, a small window was opened through the neck muscle and the skull near the end of elastic extension of hyoid bone. A small hole was then made in the cochlear dome. The cochlea was pulled out with a fine hooked wire. The removed cochleae were confirmed by visual inspection under a dissecting microscope. After cochlear removal, a recovery period on a heat pad was set over night, after which the birds were put back into the cage.

### Song recording and analysis

After each bird was individually housed in a sound-proof chamber, undirected singing was recorded once every 1–2 months using Sound Analysis Pro software, version 1.04^48^. Crystallized songs within a day were randomly selected. Analysis of syllable acoustic structure was performed using the Avisoft Saslab (Avisoft Bioacoustics). Low and high frequency noise <0.5 and >19 kHz, respectively, were filtered from the recordings, and calls were eliminated from the dataset.

A song was defined as the continuous production of syllables (phrases), followed by at least 200 ms of silence. A phrase was defined as a cluster of repetitive syllables (Fig. 1a). Analysis was performed on an average of 22.6 songs from several days per season. Syllables, the smallest unit of the song, were segmented using the Avisoft Saslab software. The separated syllables were manually double-checked for the precision of syllable separation. To measure the complexity of song syntax as a sequence of phase transition, consistency and linearity of phrase sequences, and its entropy of a first-order, Markov models were calculated as previously described^49,50^. On average, 2,576 syllables and 152.8 phrase transitions per bird were used for the analyses. For representation of phrase transition diagrams (Fig. 5a), first, the number of phrase transition types was determined and divided by the total number of all transitions to obtain a probability ranging from 0 to1 for each transition type. These probabilities were then graphed in a sequence transition diagram, where thicker and darker-colored arrows indicated more frequently produced phrase transitional probabilities^51^.

### Statistical analyses

To compare the number of syllables or phrases and the sequence of phrase transitions between intact and deafened birds and between the two years (the 1^st^ year versus the 2^nd^ year), the mean values were compared using ANOVA followed by post hoc tests with Holm’s correction. All tests were two tailed, and significance level was set at *P* < 0.05 for all comparisons. PCA of the five syllable measurements, (1) syllable duration, (2) inter-syllable interval, (3) fundamental frequency, (4) bandwidth, and (5) entropy, was performed using the prcomp package in the statistical software R (http://www.r-project.org/; R Core Team, 2013; R Foundation for Statistical Computing), to investigate the difference of phrase types between the intact and deafened birds. Sampled from each bird were 8–15 phrase types (20 syllables per phrase were used for analysis).

### Data availability

The authors confirm that all of the data underlying the reported findings are included in the article. All raw data are available from the authors upon request.
